# IgG4-Related Disease with Selective Testicular Involvement- A Rare Entity: Case Report with Review of Literature

**DOI:** 10.5146/tjpath.2020.01493

**Published:** 2021-01-15

**Authors:** Asbah Shams, Abhijit Das, Madhu Sinha, Natasha Gulati, Man Mohan Mehndiratta, Manish Kaushik, Puneet Gupta

**Affiliations:** Department of Pathology, Janakpuri Super Speciality Hospital Society, New Delhi, India; Department of Neurology, Janakpuri Super Speciality Hospital Society, New Delhi, India; Department of Surgery, Guru Gobind Singh Government Hospital, New Delhi, India; Department of Cardiology, Janakpuri Super Speciality Hospital Society, New Delhi, India

**Keywords:** IgG4-related disease, Testis, Plasma cells, Fibrosis

## Abstract

Immunoglobin-G4 related disease (IgG4-RD) is an auto-immune inflammatory condition where patients present with a tumour-like mass that shows infiltration by plasma cell and subsequent fibrosis. It is a systemic condition that primarily involves the salivary glands, pancreas, kidneys, aorta, and retroperitoneum amongst other organs. Testicular involvement is a rare occurrence in this disease entity. A 55-year old male patient presented with the complaints of pain and swelling in the right scrotal region. Right-sided orchidectomy was carried out which on histopathology showed features suggestive of IgG4-RD which was later confirmed on immunohistochemistry. Whole body MRI revealed that no other organ was involved in the disease process in this patient. IgG4-RD has a variable clinical course and considerable overlap with its differentials. Imaging studies and serum IgG4 levels are neither confirmatory nor customarily diagnostic in every case. The only confirmatory diagnostic investigation is histopathological examination, which shows infiltration of IgG4+ plasma cells and fibrosis in the involved tissue. Whenever a mass-forming lesion with typical histomorphological features is encountered with involvement of multiple organs/anatomic sites, IgG4-related disease should be considered among the differentials, and clinicians of all disciplines should be familiar with this disease entity.

## INTRODUCTION

IgG4-related disease (IgG4-RD) is a systemic, autoimmune, inflammatory condition, characterised by fibrosis of the affected organ and its infiltration by IgG4+ plasma cells, forming a tumour-like mass (1). It primarily involves the pancreas, salivary glands, kidneys, aorta and retroperitoneum amongst other organs (2). Testicular involvement is rare in this disease entity. We report a case of a 55-year old male, who presented with right-sided scrotal pain and swelling of short duration which progressed to a hard testicular lump. An orchidectomy followed and upon histopathological examination of the specimen, a diagnosis of IgG4-RD with solitary testicular involvement was rendered. Awareness of this clinical entity is imperative since it is steroid-responsive and early diagnosis and management can prevent undue morbidity.

## CASE REPORT

**Figure 1:**
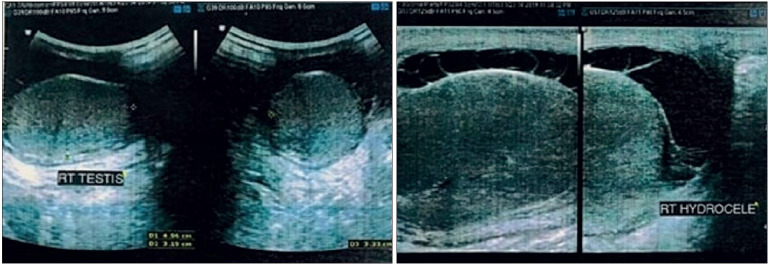
Ultrasound of scrotum shows both testes normal in size, shape and echotexture. Right scrotal sac shows mild free fluid with multiple fine internal septations.

A 55-year old diabetic male presented with complaints of pain and swelling in the right scrotal region along with fever for five days. Ultrasound of the scrotum revealed fine internal septae and free fluid in the right scrotal sac along with features of right-sided epididymitis and funiculitis. The testes on both sides were apparently normal on ultrasound (Figure 1). Consequently, in view of the ultrasound findings and clinical examination, the patient was diagnosed with right-sided acute epididymo-orchitis with localised cellulitis. Injectable broad spectrum antibiotics were started along with other supportive measures but the swelling remained persistent with conservative management. On clinical suspicion of pyocele, the patient was scheduled for incision and drainage. Intra-operatively it was noted that there was involvement of tunica albuginea by the inflammatory process as well as exposure of testicular pulp tissue. In consideration of necrotic testicular tissue, a suspicion of malignancy was raised and patient was later planned for right orchidectomy and the specimen was submitted for histopathological examination.

**Figure 2:**
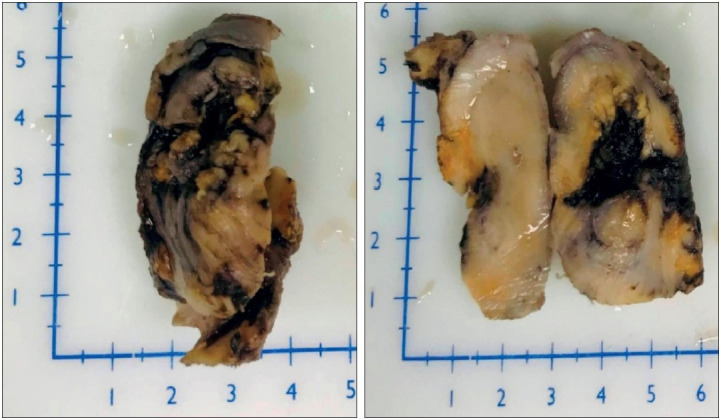
Right-sided orchidectomy specimen, grossly and on cut section, showing central necrotic area and peripheral whitish (fibrotic) area.

**Figure 3:**
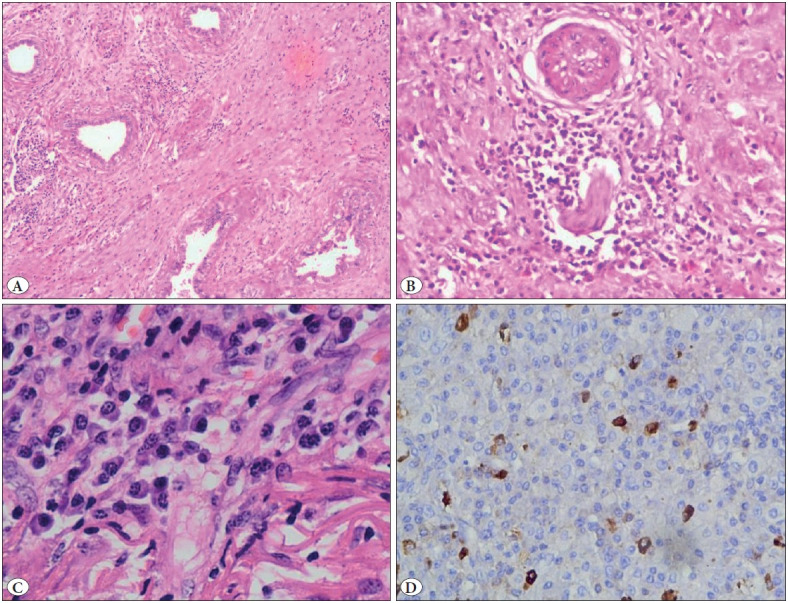
**A)** Dense lymphoplasmacytic infiltrate along with fibrosis in the interstitium distorting the normal testicular parenchyma (H&E; x20). **B)** Obliterative phlebitis (H&E; x100). **C)** Rich plasma cell infiltrate (H&E; x400). **D)** Immunohistochemistry for IgG4+ plasma cells (IHC; x200).

On gross examination, the specimen measured 5x4x3 cm. It consisted of the pulp of testes and epididymis, which on cut section showed a central tan-coloured necrotic area and peripheral whitish/fibrotic area (Figure 2). Microscopic examination revealed distorted testicular parenchymal tissue with peritubular storiform fibrosis and a rich plasma cell infiltrate. A focal area of epididymal tissue and a separately lying tunica albuginea was also seen with marked fibrosis and chronic inflammatory infiltrate, predominantly comprising of plasma cells (Figure 3). As histomorphological features were highly suggestive of IgG4-RD, immunohistochemistry for IgG4-positive plasma cells was carried out which revealed > 50 IgG4+ plasma cells per high-power field. Serum IgG4 was elevated (4.67 g/L; normal: 0.03-2.0 g/L). Hence the diagnostic criteria for IgG4-RD (3) were met and the diagnosis was confirmed.

Thereafter a comprehensive whole body work-up including whole body MRI was performed to determine the involvement of other organs but no systemic involvement could be ascertained. Furthermore, his liver, kidney and thyroid function tests as well as lipid profile and serum amylase and lipase were found to be within the normal range. Hence, it was concluded that this was a case of IgG4-related disease where the solitary site of involvement was the right testicle. The patient is doing well on follow up.

## DISCUSSION

IgG4-RD can present with lesions in a variety of anatomic sites, either synchronously or metachronously, but is shown to have similar histomorphology which is characteristic of the disease (4). Observed to be a disease of age fifty years and above, it is more commonly seen in men than in women, especially in disease affecting the kidney, pancreas and retro-peritoneum (1,5,6).

The etiology of IgG4-RD is unclear. Non-HLA genes like cytotoxic T-lymphocyte antigen-4 (CTLA-4) and tumour necrosis factor-α have also been implicated. T-follicular helper cells along with T-follicular regulatory cells help in B cell differentiation and class switching, resulting in proliferation of IgG4-secreting plasmablasts and long-lived plasma cells. CD4-positive T cells are central to the disease. Their clonally expanded population is seen in both peripheral blood and fibrotic lesions and shows active involvement in development of fibrosis. The T helper type 2 (Th2) pathway, which is responsible for eosinophilic infiltration by IL-5 release, is no longer considered central to disease pathogenesis (1,5,7).

The two consistent clinical findings are tumefactive lesions and allergic manifestations like atopy, eczema, asthma and peripheral blood eosinophilia (1,5–7). The most commonly reported organ to be involved in IgG4-related disease is the pancreas (8,9). The involvement of salivary glands by IgG4-RD is common and may be in the form of Mikulicz disease or Kuttner tumour. Various other organs associated with IgG4-RD are the retroperitoneum, kidney, thyroid, aorta, lung, lymph nodes, prostate and orbit. In kidneys, along with hydronephrosis, tubulointerstitial nephritis and membranous nephropathy have also been reported (5,8). Thyroid gland affected by IgG4-RD shows extensive fibrosis which may extend to extra-thyroidal tissues. Additionally, inflammatory pseudotumour and multifocal fibrosclerosis of the orbit, lacrimal gland, sinuses, and respiratory tract have been described (7).

Intrascrotal involvement by IgG4-RD has been reportedly limited to para-testicular structures, mostly in the form of a pseudotumour. Most of these cases had systemic disease with more than one organ involvement (10–13). Wenniger was the first to report testicular involvement in IgG4-RD in a patient with IgG4-related pancreatico-biliary disease and retroperitoneal fibrosis (14). Tokura and colleagues encountered a case of hydrocele testis, which on histopathology showed features of vaginalitis (15). Another study reported a case of IgG4-RD with selective testicular involvement having dense lymphoplasmacytic infiltrate around seminiferous tubules and IgG4 cell count 120-130/hpf. However, serum IgG4 levels were within normal limits (16).

**Figure 4:**
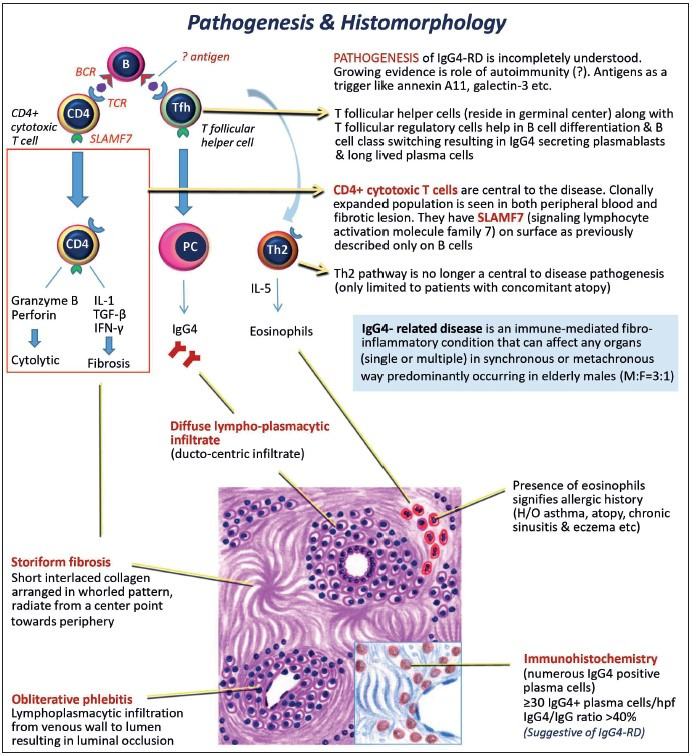
Schematic representation to describe pathogenesis and histomorphological features of IgG4-RD.

The gold standard diagnostic modality for this entity is histopathological examination. Diagnosis requires elevated IgG4+ plasma cells in the tissue and two out of these three characteristic histo-morphological features (Figure 4): 1) dense infiltrate of lymphocytes (predominantly T cells) and plasma cells, 2) storiform fibrosis, 3) obliterative phlebitis. IgG4+ plasma cell infiltration as shown by immunohistochemistry (more than 10 IgG4+ plasma cells per high power field) is considered less useful than the IgG4+ /IgG plasma cells ratio (cutoff>40%) (17). To establish the disease, in an organ/site not known to be previously involved by IgG4-RD, the following diagnostic criteria are set up: 1) typical histomorphology (as previously described) along-with high IgG4 + plasma cells in tissue and high IgG4/IgG ratio, 2) elevated serum IgG4 levels, 3) adequate response to steroid therapy, 4) involvement of other organs/sites with IgG4-related disease (10).

Elevated serum IgG4 levels (>135 mg/dl) are not mandatory for diagnosis but have shown consistent association with disease activity and show suppression after steroid therapy. IgG4-related disease responder index (RI) is a recently devised measure to ascertain the disease activity and monitor relapses during therapy. The use of imaging studies like CT scan, MRI and FDG-PET scan (18F-fluorodeoxyglucose positron emission tomography) is common for disease evaluation and depends on the organ involved and availability. Treatment with glucocorticoids is considered the first-line of management in patients with active disease. Response is prompt and is noted within days to weeks while remission is usually achieved within few months. IgG4-RD has been shown to have a chronic relapsing course and monitoring of disease activity is hence of prime importance. Recently, rituximab was introduced for treatment of this disease entity and shows promise (18).

In conclusion, IgG4-RD can present as a tumour-like mass in a number of organs/anatomic sites. It may stay indolent for years or develop organ damage in a short period of time. Imaging studies cannot reliably distinguish between this entity and malignancy. Serum IgG4 levels may not be elevated in every case but they still serve as a marker for disease activity. Since the span of this illness encompasses a number of medical specialities, familiarity with this entity is imperative and it should be considered in the differential of any mass-forming lesion that shows typical histomorphology of this disease.

## CONFLICT of INTEREST

The authors declare no conflict of interest.

## FUNDING

The authors received no financial support for the research, authorship, and/or publication of this article.
